# Large Improvements and Future Outlook

**Published:** 1920-02-07

**Authors:** 


### A YEAR'S WORK AT THE LEICESTER ROYAL INFIRMARY.

### Large Improvements and Future Outlook.

"We are fortunate, inasmuch as the signals of distress sent out by voluntary institutions have not appeared in Leicester," said Mr. J. G. Pickard, Chairman of the Finance Committee of the Leicester Royal Infirmary, when the report for the past year was presented.

The report indicates that, owing to the scarcity of labour and other difficulties, progress in connection with the building of the new orthopædic out-patient department and the pathological laboratories has been slow; indeed, for several weeks one of those distressing trade difficulties as to which class of workmen should do certain work has arisen and kept work at a standstill. The Mayor of Leicester (himself a trades' union official) had unsuccessfully endeavoured to get the parties together, and wrote that it appeared to him "little short of a public scandal that important work should be held up without anyone being made to get matters settled; human life might be sacrificed to the petty jealousies of men who ought to know better."

The military occupation of wards ceased in February last, 2,790 wounded soldiers having been admitted. Since then discharged soldiers to the number of 314 have been received. The report refers with satisfaction to the provision of £5,000 towards the erection of the new orthopædic department by the Freemasons of Leicester, in recognition of which the larger contributors have been elected to Life Governorships of the Institution, including the Provincial Grand Master, Mr. Edward Holmes. Donors of lesser amounts are also recognised. Munificent contributions of £20,000 by the executors of the late Mr. T. G. Langham, to erect a memorial to his memory, and £10,000 from the Royal Red Cross Society were recorded with satisfaction. It was mentioned that the site area of the Institution has been increased by considerable purchases of surrounding areas.

### The Work of the Year.

Four thousand four hundred and forty-two in-patients have been received—an increase of 432 on the previous year. The average daily number of beds occupied was 250;

**A Year's Work at the Leicester Royal Infirmary**—(*cont.*).

approximately eighteen patients passed through each bed—a very high average. The average stay per patient was about three weeks. To prevent discomfort and hardship in certain cases, owing to such speedy discharge, the need for increased accommodation is evident. The out-patient department showed a 23-per-cent. increase of patients during the year, and a total of 36,184. There is still overlapping of medical service, and it seems desirable that insured persons attending the out-patient department should receive supplies of drugs and dressings which may be necessary for their treatment from sources from which provision is made under the National Health Insurance. In the casualty department 59,000 attendances were recorded, as compared with 44,600; and in the orthopædic department the number reached approximately 25,000, as compared with 15,524; the latter increase recalls the need for the settlement of the building difficulties already referred to. The pathological department dealt with 6,000 examinations and reports, as compared with 3,600. *X*-ray cases numbered 4,500, as compared with under 4,000, and Dr. Astley Clarke indicated that for a third time in recent years reconstruction of this department and re-equipment with the newest appliances are desirable. All the above departments worked co-ordinately in the relief of sickness and disease. The higher the efficiency, however, the greater the cost, but the more effective the ultimate result.

Income and Expenditure.

The income of the Institution for the year was approximately £47,000 and the expenditure £49,610. Donations totalled £6,000, as compared with £4,100 in the previous year. The Hospital Sunday Fund showed an increase of £500 on the year, and the receipts from the Saturday Hospital contributions advanced from £17,900 to close on £22,000. A generous response was made by the workpeople to the appeal for a contribution of twopence instead of one penny per week. Legacies showed their variability by totalling a little over £2,000, a sum which was applied in the purchase of adjoining property, by which means it is income-producing. The cost of drugs and dressings showed an advance of £640, but the Institution is now well stocked. Salaries and wages show an increase of £2,700, due to a revision of the wages standard of the Institution to correspond with present-day needs. The Board indicates an increased cost during the current year. The salaries of sisters and nurses were once again increased as from January 1, and will add £800 to the cost of the Institution. A well-paid staff makes for efficiency, and means much to the comfort of the patients, and the Board have no doubt that the increases now granted are justified and merited.

The current year gives promise of much activity, and the Board invite the fullest confidence of the contributors to the consideration of the building scheme now proposed—namely, the erection of a new wing containing approximately 100 additional beds and the enlargement of the Nurses' Home. The year 1921 marks the 150th anniversary of the Institution, and it is hoped the occasion may be made memorable by the organisation of a great movement for the development and extension of the Institution.

The Report having been adopted on the motion of the Chairman, Mr. T. Fielding Johnson, junr., and tribute having been paid to the work of the honorary medical and surgical staff, the resident medical staff, and the matron and nursing staff, a resolution was thereupon submitted and carried unanimously approving of the Board's building scheme for the immediate erection of a new wing and the enlargement of the Nurses' Home, at an approximate cost of £50,000, provided the necessary funds were forthcoming, and inviting the honorary medical and surgical staff to meet the architect to discuss the proposed plans so that the buildings could be as complete and perfect as possible.

